# Stage 2 Registered Report: How responsibility attributions to self and others relate to outcome ownership in group decisions

**DOI:** 10.12688/wellcomeopenres.17504.2

**Published:** 2022-04-11

**Authors:** Matt Jaquiery, Marwa El Zein

**Affiliations:** 1 Department of Experimental Psychology, University of Oxford, Oxford, UK; 2 Institute of Cognitive Neuroscience, University College London, London, UK; 3 Adaptive Rationality Center, Max-Planck for Human Development, Berlin, Germany

**Keywords:** Outcome ownership, group decisions, responsibility

## Abstract

**Background:** Responsibility judgements have important consequences in human society. Previous research focused on how someone's responsibility determines the outcome they deserve, for example, whether they are rewarded or punished. Here, in a pre-registered study (Stage 1 Registered Report: https://doi.org/10.12688/wellcomeopenres.16480.2), we investigate the opposite link: How outcome ownership influences responsibility attributions in a social context.

**Methods:** In an online study, participants in a group of three perform a majority vote decision-making task between gambles that can lead to a reward or no reward. Only one group member receives the outcome and participants evaluate their and the other players' responsibility for the obtained outcome.

**Results:** We found that outcome ownership increases responsibility attributions even when the control over an outcome is similar. Moreover, ownership had an effect on the valence bias: participants’ higher responsibility attributions for positive vs negative outcomes was stronger for players who received the outcome. Finally, this effect was more pronounced when people rated their own responsibility as compared to when they were rating another’s player responsibility.

**Conclusions:** The findings of this study reveal how credit attributions can be biased toward particular individuals who receive outcomes as a result of collective work, both when people judge their own and someone else’s responsibility.

## Introduction

How we judge people’s responsibility for the outcomes of their actions has important consequences in our society. This is true for our own responsibility as well as others’. Responsibility judgements are tightly related to whether people get rewarded or blamed for the actions they make ^1, 2^, which is crucial for the maintenance of a cooperative and fair society. In many everyday situations, whether in the workplace or with our family and friends, responsibility for outcomes is shared among several individuals because these outcomes stem from collective decisions. Collective decisions can reduce the burden of individual responsibility ^3– 5^, because people feel less responsible when performing an action as a group than when acting alone ^6– 12^.

While collective decision-making reduces overall feelings of responsibility, fluctuations in feelings of responsibility are also affected by the outcome of a decision. People tend to attribute higher responsibility to themselves for positive as compared to negative outcomes. This is known as the **self-serving bias**, where people claim more credit for positive events, while they duck responsibility for negative events ^6, 9, 11, 12^. This ‘valence bias’ however does not seem to be purely selfish: it also appears when people judge their group’s or another person’s responsibility ^13, 14^. For example, in the context of advising, people exhibit an **‘other-serving’ bias** in which they tend to credit more than blame an advisor ^13^. In line with this, people also tend to attribute more effort for higher rewards when judging someone else’s effort as compared to their own effort ^15^.

In individual contexts, the rewards naturally seem to belong to the solely responsible person producing a positive or negative outcome. In a group decision where responsibility is shared, however, the outcome may be shared among group members or given to one particular group member (such as a group leader, or the group’s representative, or to a particular person the collective decision has been made for). Responsibility underlies ownership: control and intent of an action, which are directly associated with responsibility attributions ^1, 2^, also predict whether a person is perceived as the owner of an object ^16^. Moreover, a person is attributed outcomes based on how much they ’deserve’ that outcome, an effect referred as the ’entitlement effect’ ^17^. If attributions of responsibility predict ownership, is the opposite true? Something that is owned does have a special value in the eyes of its owner. This has been particularly demonstrated with the ’endowment effect’ where participants value more positively and prefer to keep goods that are given to them ^18^. Ownership could, in addition to changing the value of the owned outcome, change the sense of responsibility for that outcome. This change in responsibility as a consequence of ownership would also be consistent with the ’Just World Hypothesis’ ^19^ in which people retroactively ascribe responsibility to people for the situations they are in.

**Figure 1.**
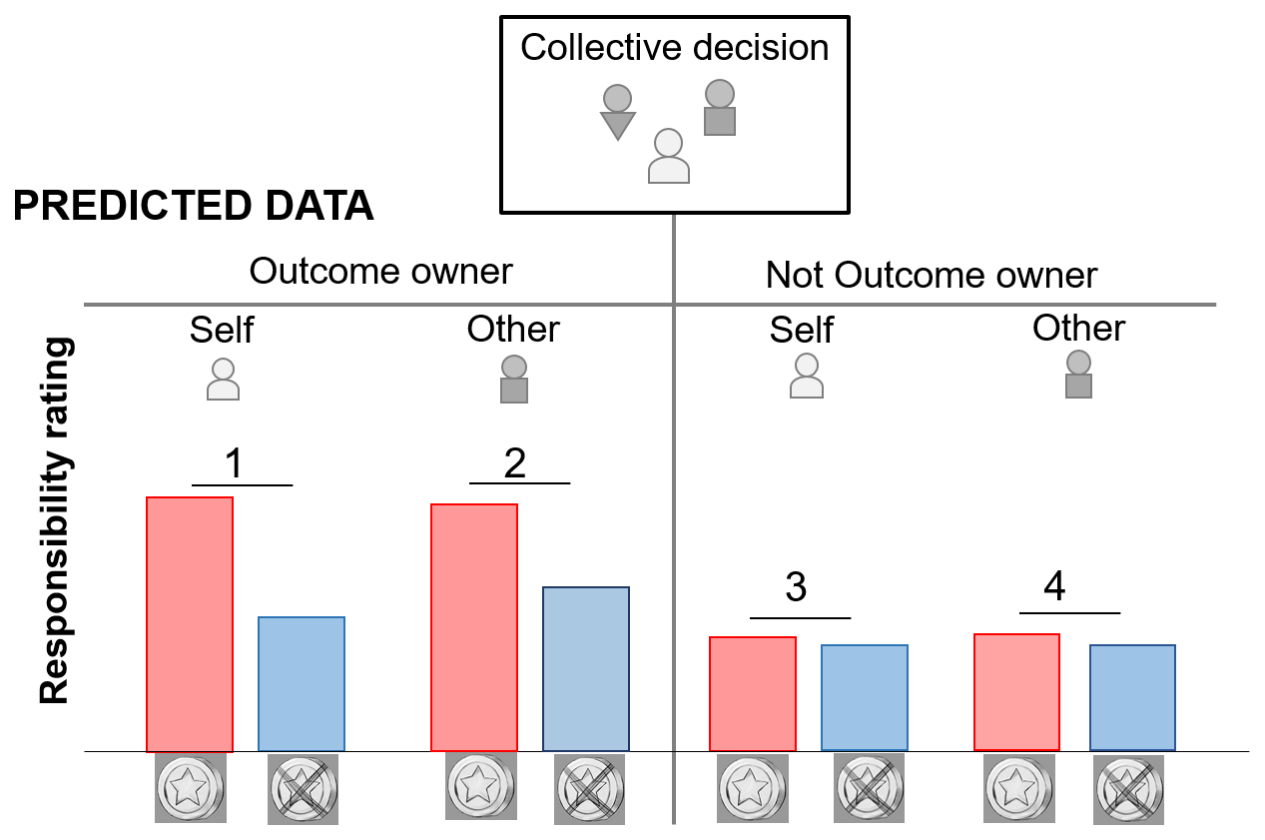
Expected responsibility ratings when people judge their own responsibility (Self) and the responsibility of another group member (Other). The ownership bias predicts a main effect of ownership where responsibility rating is higher when an outcome is owned vs not owned. The valence bias predicts a main effect of outcome valence with higher responsibility for reward vs no reward (in 1,2,3,4). An interaction between ownership and outcome valence is predicted such as the valence bias is stronger when the rated person owns the outcome (1,2) vs do not own the outcome (3,4). Finally, an interaction between ownership, outcome valence and the rated player (self/other) is expected such as the valence bias effect is the strongest when a) the outcome is owned and b) the rating concerns the self ( 1 > 2 > 3 and 4).

Here we would like to address 1) whether outcome ownership changes attributions of responsibility, and 2) whether the ‘valence bias’, i.e., increased responsibility for positive versus negative outcomes - that appears both when judging one’s own (self-serving bias) and another person’s (other-serving bias) responsibility - depends on the judged person being the owner of the outcome or not. We investigate this question in a group decision-making context, where only one member receives the outcome in each round. Participants perform an online task where they make collective decisions through majority votes, then one member of the group receives the outcome: either a reward or no reward. Finally they rate the responsibility of all group members for the positive or negative outcome. This paradigm allows us to address both questions stated above: 1) by investigating whether responsibility attribution increases when a group member receives the outcome versus does not receive the outcome, although the control over the outcome is exactly similar – referred to as the ‘ownership bias’. 2) By checking whether the valence bias of higher responsibility ratings for positive outcomes depends on whether the judged person receives the outcome of the group decision or not. In other words, in this second point, we aim to answer the question: do people exhibit the same valence bias when judging the responsibility for an outcome that is attributed not to them, but to another member of their group? Do they exhibit the same valence bias when judging the responsibility of another group member who received vs did not receive the outcome? (See Figure 1 for the predicted effects)

The results of the study allow the assessment of the link between outcome ownership and responsibility judgements, and comparison of how the valence bias for self and other is affected by outcome ownership. It helps identify biases in group responsibility attributions based on how the outcome is distributed.

The literature predominantly focuses on questions such as ’who deserves a specific positive or negative outcome based on contributions and actions?’. However, it is often the case that specific people, powerful leaders for example, receive outcomes for actions they were probably only partially or not responsible for. The work here investigates how observing a person getting an outcome can change responsibility attributions, possibly explaining how particular people may end up receiving all the credit for a collective work, both in their own eyes and others’.

## Methods

### Materials

Experiments were custom-written in HTML / CSS / JavaScript using the jsPsych framework ^20^ and undertaken by participants over the internet using their own devices. The experiment had to be performed on a desktop using a recent Google chrome or mozilla firefox browser. A demonstration version of the experiment is available at https://tinyurl.com/r-by-r-3/ATTRRESP/?demo=Y

### Procedure

The entry point to the study was through recruitment on the Prolific ( https://prolific.ac/) participant recruitment platform. The study was approved by the UCL Research Ethics Committee as Project ID Number: 5375/001. The only eligibility criterion was that participants have to be aged 18–40years. After accepting the study, they were forwarded to the experiment website. They first read an information page including ethics and data protection information. The next page was a consent form that was written in the form of sentences next to tick boxes, participants had to tick the four boxes in order to proceed: By checking the boxes below, I agree that:

1. I have carefully read the information page.
2. I have been given contact details of the researcher to ask any question or discuss the study.
3. I understand that I am free to withdraw at any time, without giving a reason, and without incurring any penalty.
4. I am over 18 years of age.

**Figure 2.**
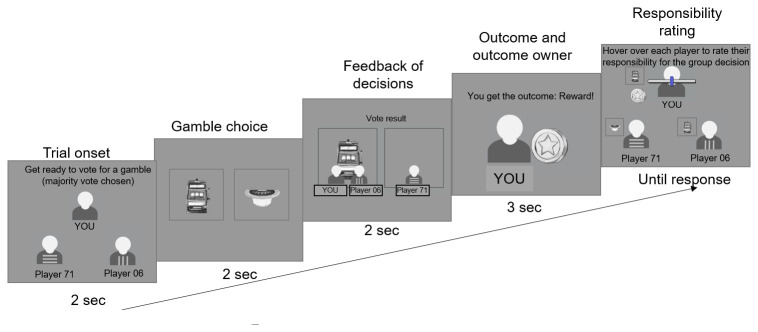
Experimental paradigm.

The experiment began with detailed instruction pages which described the structure of each round in the game, with screenshots of each stage, followed by two training trials. Once they had read the instructions and familiarised themselves with the game, participants began the main experiment, which consisted of 3 blocks of 24 trials. The 24 trials were a randomised sequence of 2 repetitions of each of the 12 unique trial types (as defined by whether the outcome is good/neutral; whether the participant is in the majority/minority; and which of the three players receives the outcome). The structure for each trial is shown in Figure 2.

At each trial, participants first saw the group for 2 sec, then saw the pair of gambles and had to pick one of the gambles by clicking on it within 2 sec. Afterwards, they saw which gamble they and the other members of their group picked for 2 sec (Here the participant and Player 71 picked the left gamble that is therefore the majority choice, i.e. the group choice, while player 06 chose the right gamble). Following this, they saw which player received the outcome, and whether that player was rewarded or not rewarded (Here, the participant receives the outcome and is rewarded). Finally, they had to rate the responsibility of each player for the outcome with no time limit except that once they finished rating the last player, the experiment continued to the next trial.

Each trial began with a display of the three players lasting 2s. This was followed by a screen lasting 2s in which the participant selected one of two gamble images. Gamble images were selected from a collection of 20 hand-drawn images of gambling devices and paraphernalia, and each pairing of images in each trial was unique. Participants were told the different gambles have different probabilities of winning and losing and that they should try and pick the one that has the higher chances of reward. However, unbeknownst to the participants, which gamble was selected had no actual influence on the outcome of the trial, which was predetermined. Once the participant selected a gamble their player icon was drawn below that option.

If the participant had not selected a gamble by the end of the 2s choice window, the rest of the trial plays out invisibly behind a warning message which told the participant that they had failed to make a choice in time. If the participant did select a gamble, the next screen was a 2s display of the votes from all three players indicating which gamble was to be selected. The gamble which received 0 or 1 votes was removed, and an 1.25s animation followed in which the gamble was allocated to one of the players and its outcome was shown (a coin for a rewarded trial or a coin with a cross through it for an unrewarded trial). The outcome then remained on the screen for 3s, showing both the valence (good/neutral) of the outcome and the player to whom it had been allocated.

Finally, all the players’ icons were restored to the screen (and next to each player was shown the gamble that they chose and their outcome if they had received the outcome on that trial). Participants assessed each player’s responsibility in the order that they picked: if they dragged the mouse toward one of the players, the slider for that player appeared and they indicated the responsibility on this slider. The responsibility rating phase lasted until the participant submitted responsibility ratings for all the players.

Once all 72 experimental trials had been completed, participants were debriefed, thanked, and returned to Prolific. Payment (£3.5) followed once all participants had completed the study and bonuses had been calculated. The bonus was given based on one randomly selected trial: if that trial was a ’reward’ trial then they were allocated the bonus of £0.5.

### Pilot experiments

The design for the study was developed over the course of three pilot experiments. The final pilot experiment used the method of the main study. We pursued two questions: 1) Does being the outcome recipient produce a difference in perceived responsibility? 2) Is the tendency to attribute more responsibility for good outcomes (valence bias) more pronounced for the outcome recipient’s perceived responsibility?

We also explored, though in a more limited manner, the extent to which an agent’s responsibility for a group decision is affected by whether they were in the majority or minority.

Pilot experiments are seldom useful for giving answers to scientific questions ^21^, although the pilot experiments reported here may be sufficiently large (N = 47, 43, and 56) to give an indication.

Pilot experiments were preregistered where possible, and analyses and links to preregistration details, methods and results are available as follows: Pilot 1 - https://tinyurl.com/r-by-r-3/analysis/pilot-1.html; Pilot 2 - https://tinyurl.com/r-by-r-3/analysis/pilot-2.html; and Pilot 3 - https://tinyurl.com/r-by-r-3/analysis/pilot-3.html. The source code, data, and analysis results for the Pilot experiments can also be found via Zenodo ^22^.

Pilot Experiments 1 and 2 used groups of 5 players (of which the participant was one), had only one player’s responsibility rated on each trial, and did not show information about which players voted for which gamble. In other respects they were similar to Pilot Experiment 3, which was used for the main study and is detailed in the Procedure section.

We observed that pilot participants consistently provided higher responsibility ratings for good as compared to neutral outcomes, both for themselves (Pilots 1 and 3) and others (Pilots 2 and 3). We also observed that the outcome recipient was seen as having a greater responsibility for the joint decision than the other players (all Pilots). Finally, we saw that the increased attribution of responsibility for good over neutral outcomes was greater when the rated person was the recipient of the outcome (all Pilots).

### Bayesian statistics

We explored the use of Bayesian statistics in the pilot experiments, but the results of the analyses proved difficult to interpret. In the power analysis and main analysis, we therefore used the frequentist ANOVA.

### Power analysis

We conducted a power analysis to determine how many participants we would need to detect effects of half the size found in the third pilot experiment. The effect for determining power was the most complicated interaction we were interested in, namely the three-way interaction which indicates a different size of effect for the valence bias increase when the outcome is owned and participants are judging the self vs another player. Power analysis was performed using custom simulation code written in R version 4.0.2 ^23^. Participants analysed in Pilot 3 were sampled randomly (with replacement) up to *N* participants. Each of the *N* participants were then used as the basis of a generative model by extracting parameters from linear regression on the participant’s pilot data. The effect size of the three-way interaction was replaced with a draw from a normal distribution with mean *E* and standard deviation equal to that observed in the pilot data (0.44). A grid search was conducted over some plausible values of *N* and *E* with 1000 simulations/cell. True (or false for *E* = 0) positive rates were calculated by running ANOVA on the data for each simulation. The power analysis indicated that 500 participants would provide around 93% power to detect an effect size of *d* = -0.07 (half of the effect size identified in Pilot 3, *d* = -0.14).

### Analysis of main experiment

First, data was excluded based on the following rules:

- All data for subsequent attempts from participants attempting the experiment more than once was dropped.
- All data for participants failing to choose a gamble on 10 or more trials was dropped.
- All data for participants who fail to use both response buttons in choosing gambles in each block was dropped.
- All data for participants whose data does not include all 72 trials was dropped.
- Trials where the participant failed to respond was dropped.
- Trials where participants stayed longer than 15 sec on the responsibility rating screen was dropped.
- All data for participants who stayed more than 15sec on the responsibility rating screen on 10 or more trials was dropped.

The data was then converted into a long format where each trial provides three observations - one responsibility rating for each player. The data was analysed (without further truncation or cleaning) using a 2 (reward vs no reward outcome) × 2 (rated player did vs did not get outcome) × 2 (rated player is vs is not participant) ANOVA with the alpha level set at .05. Participant-level data was collapsed into means for each of the six contingencies for the purposes of ANOVA.

The draft of the analysis script, as it stood at Stage 1 submission time, is available at https://github.com/mjaquiery/responsibility-by-reward/blob/2b0eb49760332f3121a9472dfd1cd2311e2121cd/analysis/main.Rmd. (archived on Zenodo ^22^).

## Results

745 participants attempted the experiment. In line with our declared exclusion criteria, we excluded 245, leaving our target sample size of 500 participants for analysis. We planned to exclude participants where: it was not their first attempt at the experiment (0); they did not choose a gamble on 10 or more trials (177); they did not use both response buttons in choosing gambles in each block (26); or their data did not include all 72 trials (62). Finally, the last 4 participants were excluded to keep to our preregistered sample size. The trial count exclusions were calculated before individual trials were removed. Individual trials were removed where: the participant failed to respond (4.97%); or the participant stayed longer than 15 sec on the responsibility rating screen (2.17%). The participants in the sample remaining after exclusions self-reported their mean age as 26.43 years (SD = 5.57; range: 18, 42). They self-reported their gender with a single character as F: 274 (55.00% female), M: 226 (45.00% male).

### Responsibility ratings

Participants’ responsibility ratings matched the pattern we expected in many respects. There was a main effect of ownership where responsibility ratings were higher for the outcome owner ( *F*(1/499) = 119.35, *p* < 0.0001, Generalized eta squared *ges* = 0.015; Mean responsibility *without* outcome = 60.42 (12.15); Mean responsibility *with* outcome = 63.93 (12.36)). This effect was present both within Self ratings (Mean responsibility *without* outcome = 55.29 (13.7); Mean responsibility *with* outcome = 60.24 (14.93); *t*(499) < 0.001, *p* < 0.0001, *d* = 0.345) and within Other ratings (Mean responsibility *without* outcome = 62.98 (14.69); Mean responsibility *with* outcome = 65.79 (14.52); *t*(499) < 0.001, *p* < 0.0001, cohen’s *d* = 0.193). We also saw the expected valence bias, with responsibility ratings being higher on trials where the outcome was a reward compared to no reward (Main effect of outcome valence: *F*(1/499) = 139.42, *p* < 0.0001, *ges* = 0.013; Mean responsibility for *no reward* outcome = 60.45 (12.35); Mean responsibility for *reward* outcome = 62.72 (11.68)). This effect was present both within Self ratings (Mean responsibility for *no reward* outcome = 55.33 (13.92); Mean responsibility for *reward* outcome = 58.58 (13.62); *t*(499) = 11.059, *p* < 0.0001, *d* = 0.236) and within Other ratings (Mean responsibility for *no reward* outcome = 63.02 (15.01); Mean responsibility for *reward* outcome = 64.79 (14.23); *t*(499) = 5.652, *p* < 0.0001, *d* = 0.122).

In terms of interactions, we expected that valence bias would be stronger when the rated person is the outcome owner, and observed the predicted outcome valence x ownership interaction ( *F*(1/499) = 109.53, *p* < 0.0001, *ges* = 0.011; Mean reward benefit *without* outcome = 0.28 (5.31); Mean reward benefit *with* outcome = 6.25 (12.84)). We observed this pattern for both Self ratings (Mean reward benefit *without* outcome = 0.34 (6.89); Mean reward benefit *with* outcome = 9.05 (15.2); *t*(499) < 0.001, *p* < 0.0001, *d* = 0.789) and Other ratings (Mean reward benefit *without* outcome = 0.24 (6.84); Mean reward benefit *with* outcome = 4.85 (13.36); *t*(499) < 0.001, *p* < 0.0001, *d* = 0.789). We also found the expected three-way interaction: the difference in reward benefit from receiving the reward was larger for Self ratings than Other ratings ( *F*(1/499) = 56.48, *p* < 0.0001, *ges* = 0.001; Mean receive reward benefit for Self = 8.72 (17.13); Mean receive reward benefit for Other = 4.61 (13.65)).

We also saw effects for which we had no formal expectations. Other ratings of responsibility were overall higher than Self ratings ( *F*(1/499) = 84.48, *p* < 0.0001, *ges* = 0.044; Mean Self responsibility = 56.95 (13.38); Mean Other responsibility = 63.91 (14.2), see Figure 4 that explains this effect by an increased rating for Other when the participant is in the minority). The other interactions were all significant, with larger effects for Self than Other for both the outcome ownership bias ( *F*(1/499) = 47.47, *p* < 0.0001, *ges* = 0.001) and the outcome valence bias ( *F*(1/499) = 30.42, *p* < 0.0001, *ges* = 0.001).

### Exploratory results: contextualising by the majority/minority status

The group decision was determined by a simple majority among three votes. The participant could thus be in the majority (i.e. endorsing the group decision) or minority (i.e. opposing the group decision). A 2x2x2x2 ANOVA, adding participant Majority/Minority status to the previous analysis, indicated that all interactions with status were significant with the exception of the three-way interaction between outcome ownership, Self/Other rating, and Majority/Minority status. Below, we explore particularly those interactions that we had identified a priori as being of particular interest.

We expected and observed a main effect of ownership where responsibility ratings were higher for the outcome owner. We observed this effect both when participants were in the Majority ( *F*(1/499) = 70.22, *p* < 0.0001, *ges* = 0.006; Mean responsibility *without* outcome = 63.8 (13.86); Mean responsibility *with* outcome = 66.38 (13.9)), and when they were in the Minority ( *F*(1/499) = 121.63, *p* < 0.0001, *ges* = 0.013; Mean responsibility *without* outcome = 57.06 (11.67); Mean responsibility *with* outcome = 61.48 (11.86)). These effects were different, in that the 2x2x2x2 ANOVA interaction was significant ( *F*(1/499) = 47.42, *p* < 0.0001, *ges* < 0.001).

We also saw that valence bias, where responsibility ratings were higher on trials where the outcome was a reward compared to no reward, was present for both Majority (Main effect of outcome valence: *F*(1/499) = 103.97, *p* < 0.0001, *ges* = 0.017; Mean responsibility for *no reward* outcome = 63.04 (14.52); Mean responsibility for *reward* outcome = 66.26 (13.52)) and Minority (Main effect of outcome valence: *F*(1/499) = 118.97, *p* < 0.0001, *ges* = 0.004; Mean responsibility for *no reward* outcome = 57.88 (11.42); Mean responsibility for *reward* outcome = 59.18 (10.94)) trials. The interaction of status and outcome valence showed these effects were significantly different ( *F*(1/499) = 17.75, *p* < 0.0001, *ges* < 0.001).

We saw, as expected, that valence bias was stronger when the rated person is the outcome owner. This outcome valence × ownership interaction was observed both when participants were in the Majority ( *F*(1/499) = 64.07, *p* < 0.0001, *ges* = 0.005; Mean reward benefit *without* outcome = 1.8 (7.76); Mean reward benefit *with* outcome = 6.05 (14.25)) and Minority ( *F*(1/499) = 110.41, *p* < 0.0001, *ges* = 0.01; Mean reward benefit *without* outcome = -1.27 (6.65); Mean reward benefit *with* outcome = 6.43 (13.31)). The three-way interaction between outcome valence, ownership, and status was significant, indicating that this effect differed by status ( *F*(1/499) = 40.32, *p* < 0.0001, *ges* < 0.001).

Finally, we saw that the difference in reward benefit from receiving the reward was larger for Self ratings than Other ratings. This effect was present both when participants were in the Majority ( *F*(1/499) = 7.07, *p* = 0.0081, *ges* < 0.001; Mean receive reward benefit for Self = 5.69 (14.79); Mean receive reward benefit for Other = 3.51 (16.74)), and when participants were in the Minority ( *F*(1/499) = 62.45, *p* < 0.0001, *ges* = 0.001; Mean receive reward benefit for Self = 11.82 (25.35); Mean receive reward benefit for Other = 5.73 (14.26)). These effects were significantly different ( *F*(1/499) = 11.35, *p* = 0.0008, *ges* < 0.001).

## Discussion

**Figure 3.**
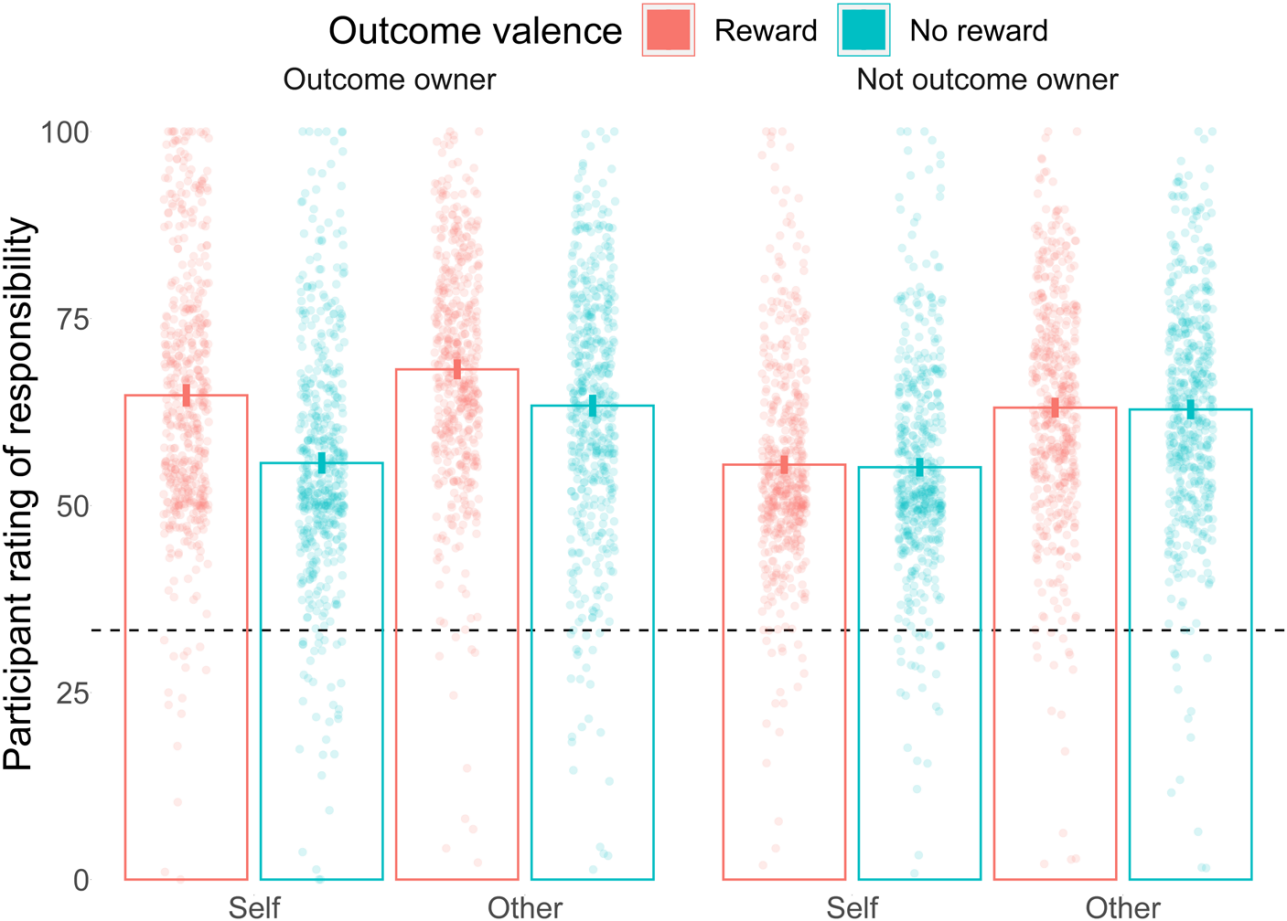
Responsibility ratings results. Mean responsibility ratings are shown for all 8 conditions. Outcome owner: when the player receives the outcome. Not outcome owner: When the player does not receive the outcome. Self: when the participant rates their own responsibility. Other: When the participant rates the responsibility of another team player. Reward is shown in red, and no reward in blue. Each point is a participant's average. The dashed line indicates 1/3rd, the expected responsibility rating if all three group members were equally responsible for the outcome. Columns show the mean values for each condition, with the error bars indicating 95% confidence intervals.

In this study, we investigated how the ownership of an outcome resulting from group decisions changes responsibility attributions. The obtained results ( Figure 3) were consistent with our predictions ( Figure 1): we found that a group member receiving the outcome of a group decision is judged more responsible than other group members who contributed equally to the decision but did not receive the outcome. We also found a valence bias with higher responsibility rating for positive vs negative outcomes that was increased by ownership, and even more so when the participants were rating their own as compared to another group member’s responsibility.

**Figure 4.**
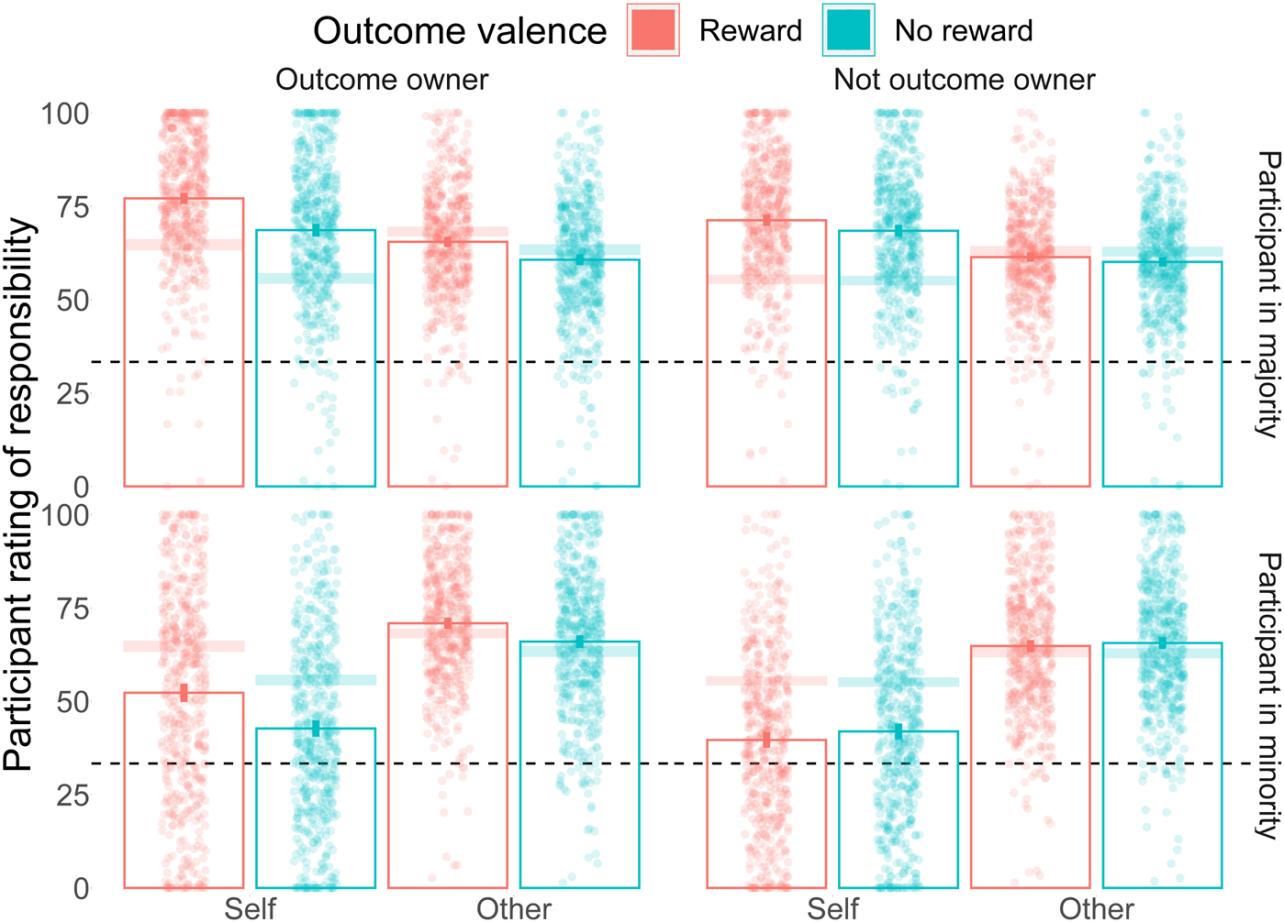
Responsibility ratings results split by majority/minority. Mean responsibility ratings are shown for all 8 conditions separately for conditions where the participant in the group majority (Upper panel) and in the group minority (lower panel). Outcome owner: when the player receives the outcome. Not outcome owner: When the player does not receive the outcome. Self: when the participant rates their own responsibility. Other: When the participant rates the responsibility of another team player. Reward is shown in red, and no reward in blue. Each point is a participant's average. The dashed line indicates 1/3rd, the expected responsibility rating if all three group members were equally responsible for the outcome. Columns show the mean values for each condition, with the error bars indicating 95% confidence intervals. The transparent bands represent 95% confidence intervals of the means collapsed across majority/minority trials.

Previous work had suggested that the establishment of ownership depends on the psychological process of responsibility attribution: intent and control over an outcome play a role in its ownership (16). Here we demonstrate the opposite direction effect: responsibility attributions depend on ownership such that even at equal levels of control over an outcome, more responsibility is given to the person who was assigned that outcome. Ownership thus may play an important role in dissociating prospective (before an outcome is shown) from retrospective (after the outcome is revealed) responsibility. When a group of people make a decision together, responsibility is shared among the group members (3–12). However, our results show that if only one of these members is given the outcome of the group decision, the post-outcome responsibility attribution will shift towards that group member. The effect is dissociated from the intent and control because participants do not know who will receive the outcome at the time of the decision, and supposedly share the control over the decision. Interestingly, participants do not seem to consider that they share the responsibility even close to equally: all responsibility ratings (on a scale of 100) were above the intuitive value of 33% if people distributed responsibility equally among the three members of the group (see Figure 3 and Figure 4). This is quite surprising as participants rated all three players at each trial and suggests that responsibility attributions do not rely on a purely additive process, making them prone to changes based on outcome valence and ownership. A limitation of our study, however, is that we did not ask for responsibility ratings before the outcome to compare shifts in responsibility from the moment of the decision to the outcome ^24^. This could have allowed us to directly compare pre and post outcome responsibility based on ownership, and to assess whether there is a specific increase for positive outcomes and/or decrease for negative outcomes from a baseline prospective responsibility level.

Still, by comparing ratings after positive vs negative outcome, we found that the ownership responsibility shift is not a simple additive process: *credit* attribution more specifically is increased for the outcome owner. These results highlight potential biases in credit and responsibility attributions to the specific people who receive an outcome resulting from a shared decision process. An award winner will be attributed more responsibility for the work done as a team than the rest of the team members. A CEO receiving praise for their companies’ achievements will be judged as more responsible than the rest of the employees who contributed equally to these achievements.

The increased credit responsibility based on ownership is more salient in one’s own judgements than those of others. Self-serving bias (6,9,11,12) and other-serving bias (13,14) thus both depend on outcome ownership, but our results provide a first direct comparison of the two and show that the self-serving bias is stronger. This may create overconfidence and over-attribution for the person receiving a positive outcome, but also suggests that one way to reduce such credit-ownership bias is to evaluate responsibility through the eyes of others. In an electroencephalography study, a brain component associated with learning (fERN) was shown to be sensitive to the difference between rewards and no rewards only for self-ownership ^25^. The observed effects on responsibility may thus also reflect the fact that participants more diligently track the difference between rewards and no rewards when the outcome belongs to them, which leads to higher biases in their responsibility judgments, but possibly also allows them to learn better from rewards. A limitation of our study is that our task afforded no genuine opportunity for learning, and so we cannot test this in our data, but this hypothesis should be tested in future research. Previous studies suggested that in individual learning, participants learn more about the consequences of their actions when they are free to choose vs instructed ^26^ and learn more from positive than negative outcomes only when they are free to choose ^27^. Thus, in addition to being in control, we predict that outcome ownership could similarly increase learning from positive outcomes.

Our study puts forward the role of ownership in responsibility attributions in group behaviour. This bias may have an interesting motivational potential for societal issues suffering from diffusion of responsibility like climate change actions. If ownership is so important for people to feel more responsible, one may think of ways to reward people for their pro-environmental actions, such as offering them reward points. ‘Owning’ reward points for their collective action would thus increase their feelings of individual responsibility which could be beneficial to reinforcing the pro-environmental action. 

## Data availability

### Underlying data

Zenodo: mjaquiery/responsibility-by-reward: Stage 1, https://doi.org/10.5281/zenodo.4452004 ^22^

This project contains the data for the pilot experiments, along with data dictionaries describing the variables. These are available in .csv format from ./data directory of the GitHub repository ( https://github.com/mjaquiery/responsibility-by-reward/tree/master/data).

Zenodo: mjaquiery/responsibility-by-reward: Stage 2 Review, https://doi.org/10.5281/zenodo.5762150 ^28^.

This project contains the actual experiment data. The Pilot 3 data dictionary is available from the GitHub repository ( https://github.com/mjaquiery/responsibility-by-reward/blob/master/data/dictionary_pilot_3.csv).

Data are available under the terms of the Creative Commons Attribution 4.0 International license (CC-BY 4.0).

### Extended data

Analysis code for experiments available here: https://github.com/mjaquiery/responsibility-by-reward/tree/master/ATTRRESP

Archived analysis code as at time of publication:

- Stage 1: http://doi.org/10.5281/zenodo.4452004 ^22^
- Stage 2 analysis: mjaquiery/responsibility-by-reward: Stage 2 Review | Zenodo ^28^

License: CC-BY 4.0
