# Supplementary material for: Stage 2 Registered Report: How responsibility attributions to self and others relate to outcome ownership in group decisions
Source: Wellcome Open Res. 2022 Apr 11;6:362. Originally published 2021 Dec 23. [Version 2] doi: 10.12688/wellcomeopenres.17504.2 (PMC8961199; doi:10.12688/wellcomeopenres.17504.2)
Supplement: Peer review history

# Peer review: Reviewer response for version 1

**DOI:** 10.21956/wellcomeopenres.19354.r47756

**Published:** 2022-03-28

**Authors:** Nura Sidarus

**Affiliations:** 1 Department of Psychology, Royal Holloway University of London, Surrey, UK

This article investigates how responsibility judgments are influenced by whether action outcomes are desirable or not, and how that effect is influenced by being the owner, or recipient, of the outcomes, as well as whether one is judging oneself or others. Pilot studies support the presence of an ownership bias, where responsibility is higher for the outcome owner than non-owners, and for a valence bias for the outcome owner, which is seen as more responsible for positive than negative outcomes, which is larger for oneself (self-serving) than for others (other-serving bias). These are very interesting research questions and methods, bridging between fields of research that refer to related concepts, combining information to arrive at new hypotheses and providing a strong way to operationalise and test the ideas.

 The use of a registered report, with transparent sharing of the details of pilot data collected and analysis already performed, is commendable, helping to clarify the research ideas and demonstrate the researchers’ strengths to competently conduct the planned research. The pilot studies provide a strong grounding for the planned study and for the detailed power analysis based on simulations to estimate the necessary sample size for the main study to test the relevant 3-way interaction.

 The final results supported the initial hypotheses, are clearly described, with clear figures, and follow the pre-registered plans. The extra analysis is clearly identified as such and highlights an interesting factor to consider in future research on shared decision-making, which is the relevance to responsibility attributions of whether one’s decision is in line or not with the majority choice. Data and analysis code are accessible. The discussion adequately interprets the findings, integrates them with previous work, and makes useful suggestions for future research.  The authors have adequately addressed previous reviewer comments at Stage 1, thus improving the final manuscript, with no further changes needed. Therefore, both the article and the materials generated by this work make a very valuable contribution to the field.

Is the work clearly and accurately presented and does it cite the current literature?

Yes

If applicable, is the statistical analysis and its interpretation appropriate?

Yes

Did the authors adhere precisely to the registered experimental procedures? If not, has an explanation been provided regarding any change?

Yes

Are all the source data underlying the results available to ensure full reproducibility?

Yes

Is the study design appropriate and is the work technically sound?

Yes

Are the data able to test the authors’ proposed hypotheses by satisfying the approved outcome-neutral conditions (such as quality checks, positive controls)?

Yes

Are the conclusions drawn adequately supported by the results?

Yes

Are the introduction, rationale and stated hypotheses the same as the approved Stage 1 submission?

Yes

Are any unregistered post hoc analyses added by the authors justified, methodologically sound and informative?

Yes

Are sufficient details of methods and analysis provided to allow replication by others?

Yes

Reviewer Expertise:

Cognitive neuroscience, sense of agency, metacognition, decision making, learning

I confirm that I have read this submission and believe that I have an appropriate level of expertise to confirm that it is of an acceptable scientific standard.

# Peer review: Reviewer response for version 1

**DOI:** 10.21956/wellcomeopenres.19354.r47757

**Published:** 2022-02-03

**Authors:** John A. Dewey

**Affiliations:** 1 Department of Psychological Sciences, University of North Georgia, Dahlonega, GA, USA

This study investigated how people experience responsibility for group decisions, using a computerized gambling paradigm. The main findings relate to how ownership of outcomes (one is said to ‘own’ the outcome when they receive the consequences) and valence of outcomes (reward or punishment) influence attributions of responsibility for the group decision. The results supported the authors’ two hypotheses. Specifically, outcome ownership tended to increase attributions of responsibility to the actor who received the consequences. Also, there was a bias to attribute more responsibility for owned outcomes with a positive valence, which was more pronounced for the self (i.e. a possible self-serving bias).

 The authors addressed the methodological concerns I raised during Stage 1. In the earlier version of the gambling task, the instructions said there was “ *no link between the type of image depicted (roulettes, dice, cards) and the actual probability to win*”. The instructions were updated so that participants would not believe their choices made no difference. My other concern was that participants might forget their choices or the choices of their group before they could judge their responsibility. The authors addressed this by adding a visual indication of each group members’ choices during the ratings.

 Overall, the methods and results are transparent, the article seems to be technically sound and makes a straightforward empirical contribution to the literature on attribution and sense of agency.

 I note that some of the effect sizes seem quite small. Even so, the idea of manipulating ‘ownership’ to motivate a sense of social responsibility seems like a promising direction for applied work.

 I only have one further suggestion. All the figures are readable but Figures 1 and 2 are noticeably sharper-looking than Figures 3 and 4. I assume the latter two were created in R. Perhaps the dpi/resolution for those two figures should be increased.

Is the work clearly and accurately presented and does it cite the current literature?

Yes

If applicable, is the statistical analysis and its interpretation appropriate?

Yes

Did the authors adhere precisely to the registered experimental procedures? If not, has an explanation been provided regarding any change?

Yes

Are all the source data underlying the results available to ensure full reproducibility?

Yes

Is the study design appropriate and is the work technically sound?

Yes

Are the data able to test the authors’ proposed hypotheses by satisfying the approved outcome-neutral conditions (such as quality checks, positive controls)?

Yes

Are the conclusions drawn adequately supported by the results?

Yes

Are the introduction, rationale and stated hypotheses the same as the approved Stage 1 submission?

Yes

Are any unregistered post hoc analyses added by the authors justified, methodologically sound and informative?

Yes

Are sufficient details of methods and analysis provided to allow replication by others?

Yes

Reviewer Expertise:

Sense of agency; control; ownership

I confirm that I have read this submission and believe that I have an appropriate level of expertise to confirm that it is of an acceptable scientific standard.
